# The electrical heart axis and ST events in fetal monitoring: A post-hoc analysis following a multicentre randomised controlled trial

**DOI:** 10.1371/journal.pone.0175823

**Published:** 2017-04-14

**Authors:** Rik Vullings, Kim M. J. Verdurmen, Alexandra D. J. Hulsenboom, Stephanie Scheffer, Hinke de Lau, Anneke Kwee, Pieter F. F. Wijn, Isis Amer-Wåhlin, Judith O. E. H. van Laar, S. Guid Oei

**Affiliations:** 1 Department of Electrical Engineering, Eindhoven University of Technology, Eindhoven, the Netherlands; 2 Department of Obstetrics and Gynaecology, Máxima Medical Centre, Veldhoven, the Netherlands; 3 Department of Obstetrics and Gynaecology, University Medical Centre Utrecht, Utrecht, the Netherlands; 4 Department of Clinical Physics, Máxima Medical Centre, Veldhoven, the Netherlands; 5 Department of Women and Child Health, Karolinska Institute, Stockholm, Sweden; University of Washington, UNITED STATES

## Abstract

**Objective:**

Reducing perinatal morbidity and mortality is one of the major challenges in modern health care. Analysing the ST segment of the fetal electrocardiogram was thought to be the breakthrough in fetal monitoring during labour. However, its implementation in clinical practice yields many false alarms and ST monitoring is highly dependent on cardiotocogram assessment, limiting its value for the prediction of fetal distress during labour. This study aims to evaluate the relation between physiological variations in the orientation of the fetal electrical heart axis and the occurrence of ST events.

**Methods:**

A post-hoc analysis was performed following a multicentre randomised controlled trial, including 1097 patients from two participating centres. All women were monitored with ST analysis during labour. Cases of fetal metabolic acidosis, poor signal quality, missing blood gas analysis, and congenital heart disease were excluded. The orientation of the fetal electrical heart axis affects the height of the initial T/QRS baseline, and therefore the incidence of ST events. We grouped tracings with the same initial baseline T/QRS value. We depicted the number of ST events as a function of the initial baseline T/QRS value with a linear regression model.

**Results:**

A significant increment of ST events was observed with increasing height of the initial T/QRS baseline, irrespective of the fetal condition; correlation coefficient 0.63, p<0.001. The most frequent T/QRS baseline is 0.12.

**Conclusion:**

The orientation of the fetal electrical heart axis and accordingly the height of the initial T/QRS baseline should be taken into account in fetal monitoring with ST analysis.

## Introduction

Fetal asphyxia is associated with severe perinatal morbidity and mortality. The cardiotocogram, a simultaneous recording of the fetal heart rate and uterine contractions, is used worldwide for fetal surveillance. However, the poor specificity of this method has resulted in increased rates of operative deliveries without a decrease in perinatal mortality or cerebral palsy [1]. ST analysis (STAN) was introduced in 1992 as a promising technique that analyses the ST segment of the fetal electrocardiogram (ECG), acquired using an invasive scalp electrode [2]. ST analysis combined with cardiotocography was reported to significantly lower the rates of metabolic acidosis [3] and operative delivery in two randomised controlled trials [3,4]. However, subsequent multicentre trials, including the recently published large American STAN trial, could not reproduce these initial findings [5–9]. Recent meta-analyses show conflicting results regarding the decrease in metabolic acidosis, which indicates the need for more research [8,10–13]. Meanwhile, Kwee et al. [14] reported that the STAN monitor gives as many ST events in cases of proven uncompromised fetal condition as in situations with deteriorating fetal condition. This is countered by the STAN guidelines that state that ST events must be ignored when cardiotocography shows a reassuring pattern. However, the high inter-observer variability in cardiotocogram interpretation makes this a highly unsatisfying strategy [15]. The correct interpretation of a method as subjective as the cardiotocogram determines whether or not to ignore the ST event or to act upon the alarm, making the success of ST monitoring totally dependent on cardiotocogram assessment.

### Background information and hypothesis

Prior to the introduction of ST analysis, the diagnostic value of the fetal ST segment was clearly demonstrated in animal studies [16–18]. Sustained deprivation of oxygen is followed by a surge of adrenalin to induce glycogenolysis, which is accompanied by an increase of potassium ions in the myocardial cells [19]. These potassium ions mainly affect the relaxation phase of the cardiac cycle and lead to an increase in the T-wave amplitude of the fetal ECG [20].

STAN uses this hypoxia-related rise in T-wave amplitude in a three-step protocol. 1. The T-wave amplitude is normalised against the amplitude of the QRS-complex (mean of 30 ECG complexes), yielding a T/QRS value. 2. A baseline T/QRS value is determined (median of at least 20 T/QRS values within 20 minutes) to gauge future T/QRS values. 3. New T/QRS values are compared to the baseline. In case a T/QRS value exceeds the baseline by 0.05, a baseline ST event is reported. Smaller exceedings of the baseline can be due to normal beat-to-beat fluctuation in the behaviour of the heart, which is unrelated to the fetal condition. With regard to the detection of rises in T-wave amplitude due to oxygen deprivation, this alarm protocol seems plausible.

The ECG recorded from the fetal scalp electrode is a one-dimensional presentation of the electrical activity of the heart. However, the propagation of electrical currents over the cardiac muscle occurs in all three spatial dimensions. The main direction of this propagation is referred to as the electrical axis of the heart. The orientation of the electrical heart axis with respect to the fetal scalp electrode hence affects the shape and amplitude of the recorded ECG. Similarly, (adult) ECG signals recorded at different locations yield different shapes and amplitudes, as already demonstrated many years ago [21].

It is known that the orientation of the fetal electrical heart axis can vary between +100 and +160 degrees in mid-term fetuses [22] and between +90 and +180 degrees in term fetuses during labour [23]. Similar inter-person variation in the orientation of the electrical heart axis is present in neonates and adults [24–27]. The STAN monitor attempts to correct for the orientation of the electrical heart axis with the first step in its protocol (normalisation). However, the propagation of the electrical currents during the contraction (QRS) phase of the cardiac cycle has a different orientation than during the relaxation (T) phase. Consequently, normalisation cannot fully compensate for inter-patient differences in the orientation of the electrical heart axis. As a result, fetuses for whom the scalp lead is almost perpendicular to the direction of propagation in the relaxation phase have a very small T-wave amplitude, and typically also low T/QRS values and T/QRS baseline. Similarly, fetuses for whom the electrical heart axis is oriented in a manner creating a propagation during relaxation almost aligned with the scalp lead, typically have a high T/QRS value and T/QRS baseline.

**Fig 1:**
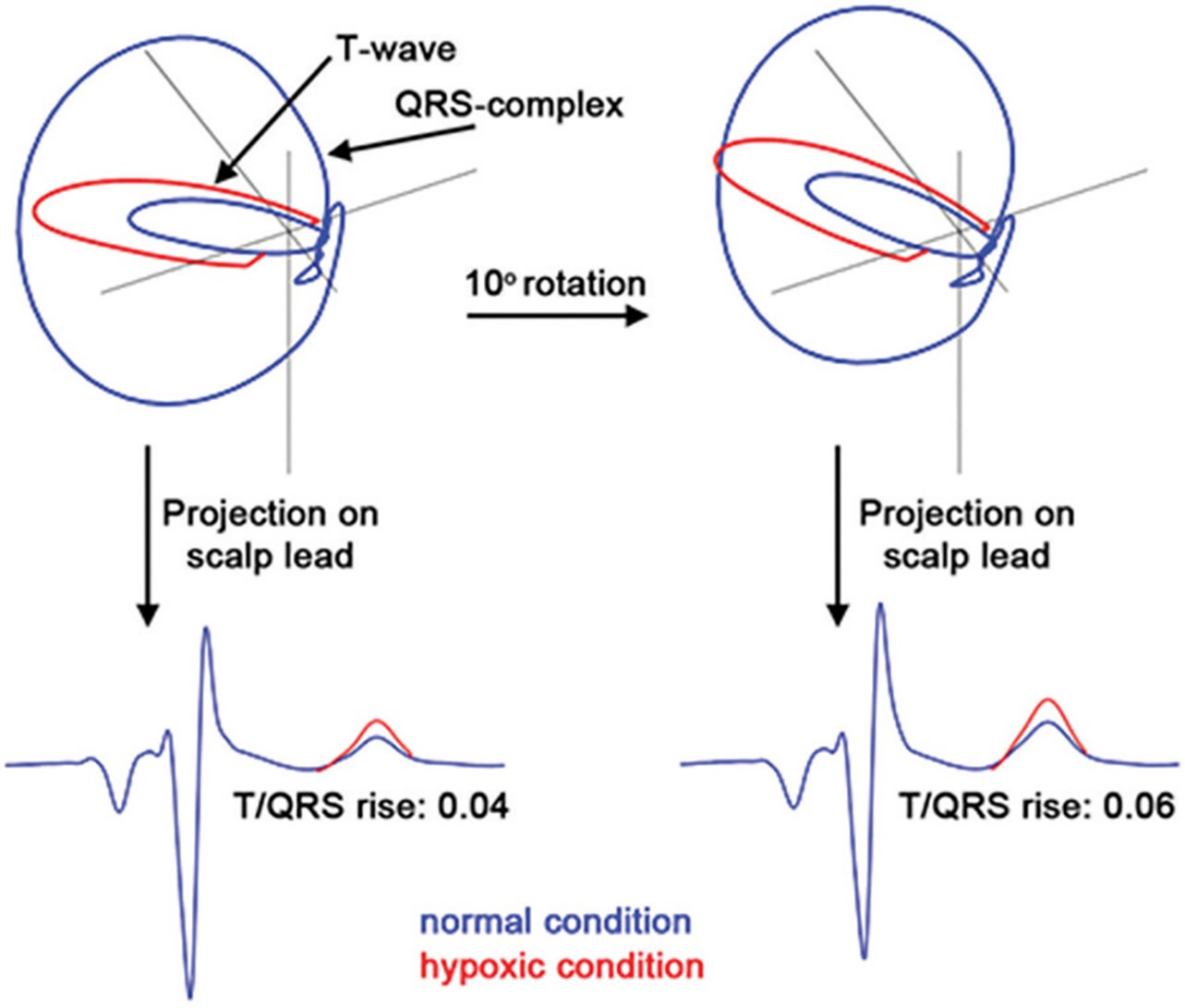
The fetal vectorcardiogram for different orientations of the electrical heart axis. In the top panels, the electrical currents within the heart during a cardiac cycle are depicted in terms of their vectorcardiogram; ventricular contraction (QRS-complex = large loop), relaxation phase (T-waves = small loop). From left to right, the entire vectorcardiogram has been rotated over 10 degrees to simulate a different orientation of the electrical heart axis. Note that these vectorcardiograms are 3-dimensional images and the 10 degree rotation was performed in 3-dimensional space. In the bottom panels, the fetal scalp ECG has been calculated by projecting the vectorcardiograms onto the scalp lead. Before rotation, the baseline T/QRS is 0.05 and the T/QRS rise resulting from hypoxia is 0.04, yielding no ST event. After rotation, the baseline T/QRS is 0.09 and the T/QRS rise resulting from the same level of hypoxia is 0.06, yielding a ST event.

When the hypoxia-induced release of potassium ions affects the electrical current in the relaxation phase in the fetuses with a low T/QRS baseline value, the absolute effect in T-wave amplitude will only be marginal as we look at it from an almost perpendicular direction. In fetuses with high T/QRS values, the rise in T-wave amplitude will be relatively large. Based on this, we hypothesise that normal fluctuations in the electrophysiological behaviour of the heart can stay below the 0.05 threshold, in case the scalp lead is oriented more perpendicular to the relaxation currents. Similarly, the hypoxia-related fluctuations in the electrical behaviour can more easily exceed the 0.05 threshold, when the alignment between the electrical heart axis and scalp lead is axial. We explain this phenomenon in Fig 1. Previously, Becker et al. [28] described that the initial T/QRS baseline is not related to the fetal condition. The incidence of ST events was stated to be related to the fetal condition [3], and therefore not related to the baseline. This is in contrast with our hypothesis that the STAN monitor will raise fewer ST events for fetuses with a low baseline, and more ST events for fetuses with a high T/QRS baseline.

This paper aims to explain how false ST events can occur, based on normal variations in human physiology. Based on this explanation, clinicians might be able to make a better informed decision whether or not to act upon a ST event in case of inconclusive cardiotocogram assessment.

## Materials and methods

We performed a post-hoc analysis with data derived from a large multicentre randomised controlled trial, the Dutch STAN trial [7]. The initial study was approved by the Institutional Review Board of the University Medical Centre Utrecht and was performed between January 2006 and July 2008. After written informed consent, women were randomised to the index group with ST monitoring (STAN S21 or S31 fetal heart monitor) or to the control group, using a conventional fetal heart rate monitor (cardiotocography). The randomisation was performed on a 1:1 basis, web-based with stratification for centre and parity. Since the trial was pragmatic in nature, there was no blinding of patients or caregivers. All gynaecologists, residents and midwives in the participating centres were trained and certified as STAN-users, and decisions were made following the STAN clinical guidelines. Fetal blood sampling was allowed, yet restricted to specific scenarios in the index group. Inclusion criteria were maternal age over 18 years, singleton pregnancy, cephalic presentation, gestational age beyond 36 weeks and an indication for internal electronic fetal monitoring. The included women were assigned to a “high-risk pregnancy” group, since they all received secondary care. In the Netherlands, “low-risk pregnancies” are monitored by midwives or general practitioners (primary care). In both groups, the umbilical cord was double clamped immediately after birth, in order to sample both arterial and venous cord blood.

For this post-hoc analysis, we included data from two tertiary hospitals: the University Medical Centre Utrecht and the Máxima Medical Centre Veldhoven, both participating in the multicentre randomised controlled trial. Anonymised information from the initial database was used for this analysis. Following consultation of the Medical Ethical Department in the Máxima Medical Centre, no separate ethical approval was warranted for this study. We only included patients from the index group (with ST monitoring). We excluded patients in whom no STAN registration was performed or no T/QRS baseline value could be determined, cases of metabolic acidosis, cases in which no blood gas analysis was performed postpartum and registrations performed in fetuses with congenital heart disease. Metabolic acidosis was defined as umbilical cord artery blood pH <7.05 and base deficit of the extracellular fluid compartment >12 mmol/l in two blood samples with a minimal pH difference of 0.03. In cases of only one blood sample or smaller differences between samples, metabolic acidosis was set as cord blood pH <7.10 and base deficit of extracellular fluid >12 mmol/l.

The initial baseline T/QRS value was determined the same way as done in the STAN monitor; as the median of all T/QRS values recorded within the first 20-minute window of the recording, that contained a minimum of 20 T/QRS values. We counted the incidence of ST events throughout the entire registration. Patients were excluded in case a STAN registration was temporarily stopped and more than one STAN file was stored for the patient. For each initial baseline T/QRS value encountered in our data set, we counted the number of patients with that particular baseline. We grouped women with the same initial baseline T/QRS value. Hereafter, we calculated the relative incidence of ST events (defined as the number of ST events per 1000 T/QRS values) as a function of the initial baseline value.

Additionally, we calculated the mean pH and mean base deficit of the extracellular fluid for all women with the same initial baseline T/QRS value. Even though our dataset entails a subset of the data used by Becker et al. [28], it needs to be confirmed that the conclusions from this study, that the height of the initial baseline is not related to fetal outcome, apply to our dataset as well.

Matlab (The Mathworks, Natick, MA) was used to perform the statistical analysis. For analysis of the baseline characteristics, mean, median, standard deviation and interquartile ranges were calculated using IBM SPSS statistics 22.0 for Mac (IBM corp. Armonk, NY, USA). A linear regression model was used to calculate the correlation coefficient for the relation between the number of ST events and the baseline T/QRS value.

## Results and discussion

Initially, 1401 patients were screened; in 273 cases ST information was missing, more than one STAN file was available for the same patient, or no T/QRS baseline value had been determined due to short duration of the measurement or poor quality of the data. These cases were therefore excluded. In addition, we excluded 11 cases of fetal metabolic acidosis. Further, no blood sample was available in 12 patients, whom were therefore excluded. In addition, 6 women gave birth to neonates with congenital heart disease, 1 labouring woman younger than 18 years and 1 prior to 36 weeks of gestation during labour were excluded. Eventually, we analysed the number of ST events in 1097 women. In this group, a total of 1.027.054 T/QRS ratios and 2066 ST events were reported. The baseline characteristics of the included women are summarised in Table 1.

**Table 1:** Baseline characteristics of the included patients.

Variable		
Centre UMCU (%)	47.4	
MMC (%)	52.6	
Maternal age (years; mean, [SD])	31.9	[4.6]
Nulliparous (%)	53.4	
Gestational age at delivery (weeks; mean, [SD])	40+0	[1+3]
Spontaneous onset of labour (%)	65.4	
Induction (%)	34.6	
Fetal blood sampling (%)	10.4	
Spontaneous delivery (%)	77.6	
Operative vaginal delivery (%)	10.1	
Caesarean section (%)	12.3	
Apgar score 1’ (median, [IQR])	9	[1]
Apgar score 5’ (median, [IQR])	10	[0]
pH arterial (mean, [SD])	7.22	[0.07]
Base deficit arterial (median, [IQR])	6	[4]
Birth weight (gram; mean, [SD])	3562	[509]
NICU admission (%)	1.7	
Medium care admission (%)	13.8	
Perinatal mortality (%)	0	

Abbreviations: UMCU; University Medical Centre Utrecht, MMC; Maxima Medical Centre, SD; standard deviation, IQR; interquartile range.

**Fig 2:**
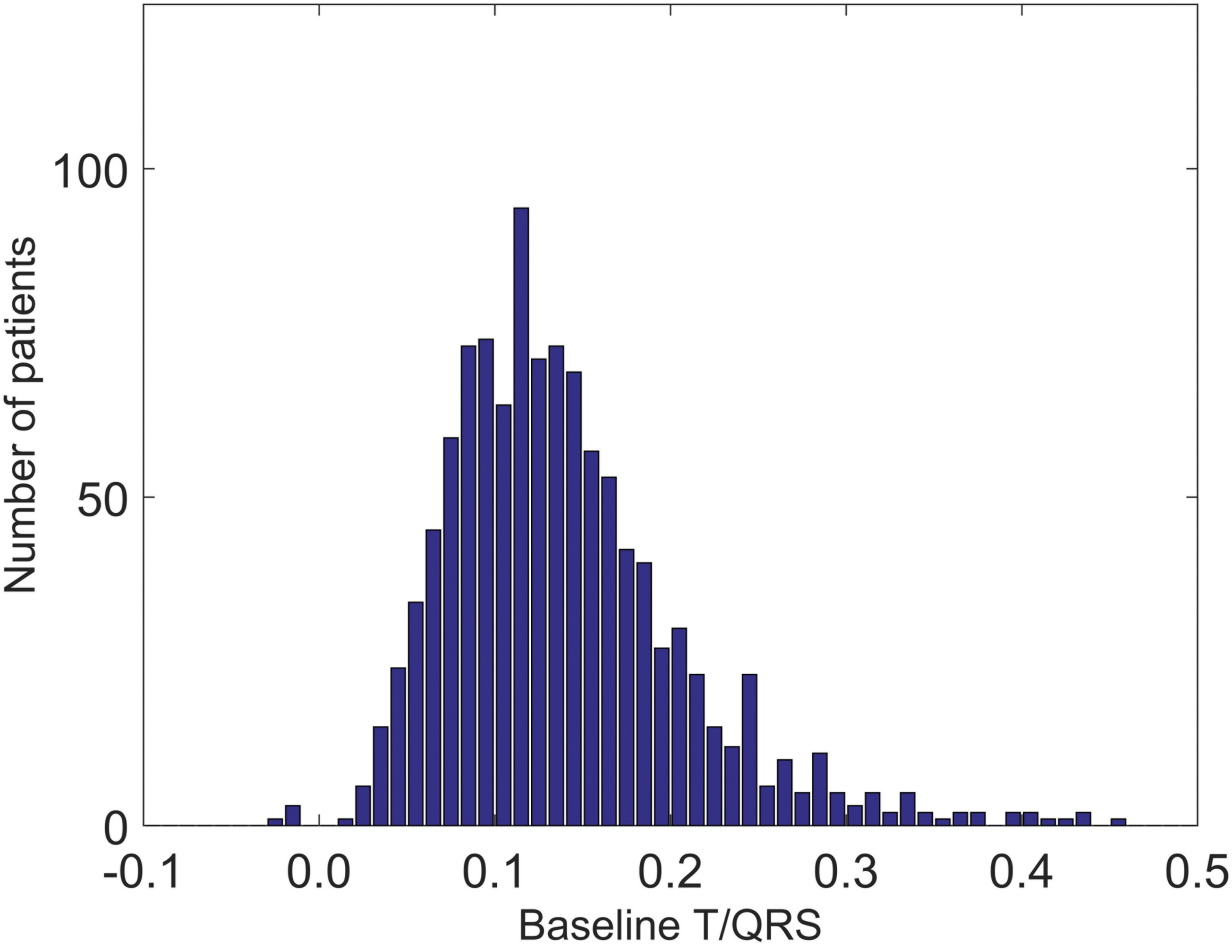
Distribution of patient across the baseline T/QRS values. For each initial baseline T/QRS value encountered in our data set, we counted the number of patients with that particular baseline, showing a non-symmetric distribution with the most frequent encountered baseline T/QRS ratio at 0.12.

**Fig 3:**
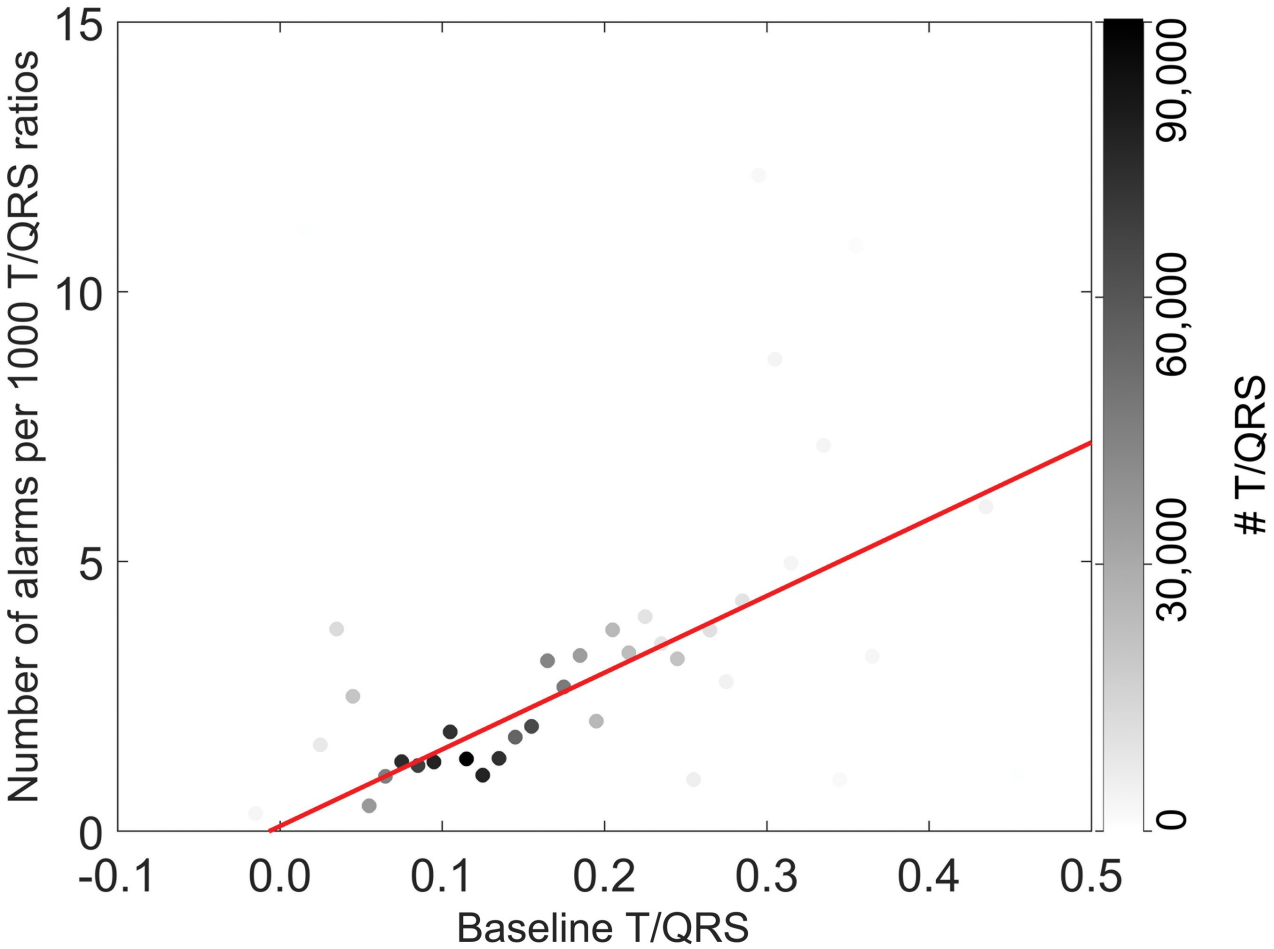
The number of ST events per 1000 T/QRS values as a function of the initial baseline T/QRS value. Cases with the same initial baseline T/QRS were grouped. The intensity of the black colour of the data points relates to the total number of T/QRS ratios that occurred in the group (right column in the graph). The red line represents a linear fit through the data points. There is an average increment of 1.42 ST events per 1000 T/QRS values for a rise of 0.1 of the initial baseline T/QRS. The correlation coefficient between data points and fit was 0.63 (p <0.001), as calculated with the linear regression model.

Fig 2 shows the distribution of patients across the various initial baseline T/QRS ratios. In Fig 3, we present the number of ST events as a function of the initial baseline T/QRS value. The results show an average increment of 1.42 ST events per 1000 T/QRS values for a rise of 0.1 of the initial baseline T/QRS. The correlation coefficient between data points and fit was 0.63 (p <0.001), as calculated with the linear regression model.

**Fig 4:**
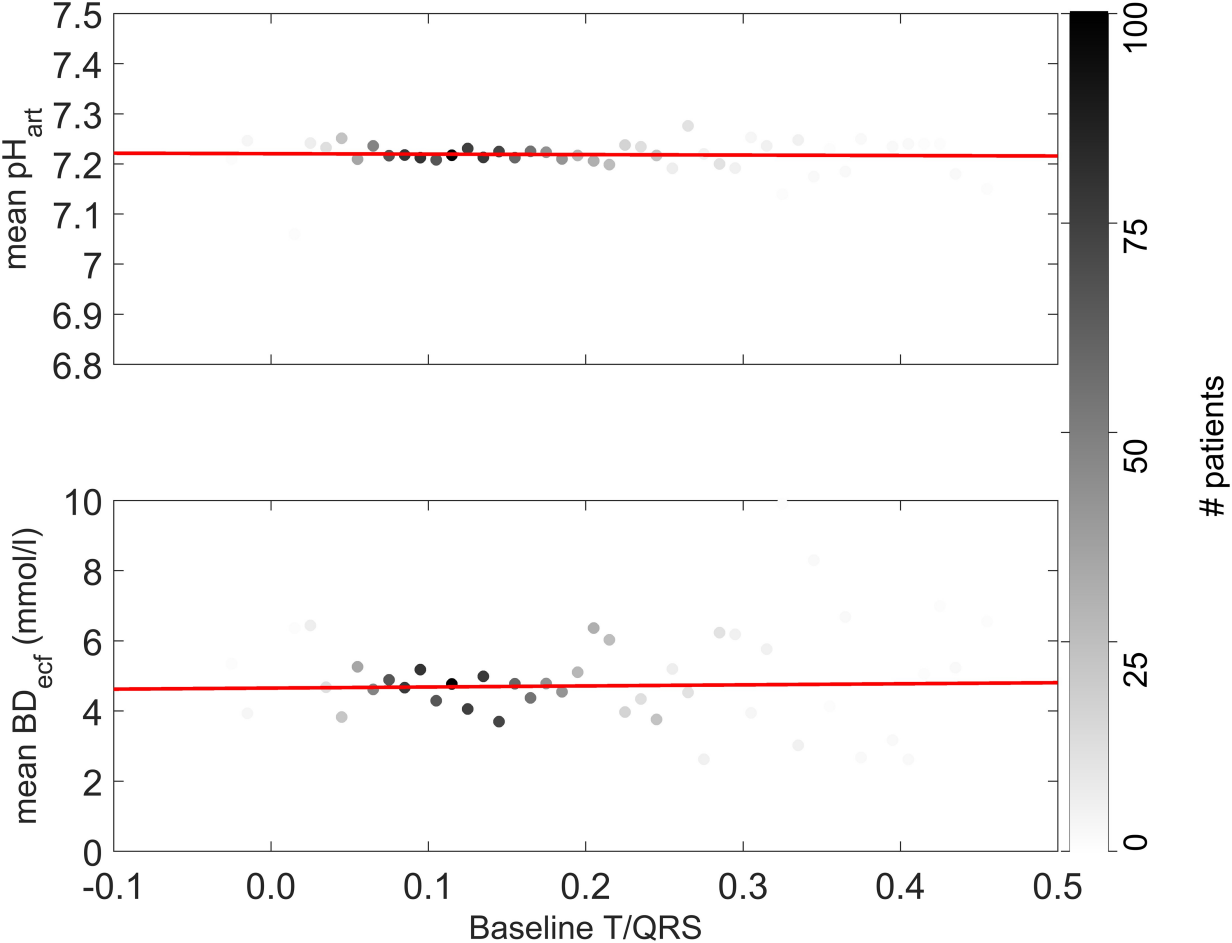
The pH of arterial cord blood and base deficit of the extracellular fluid as a function of the initial baseline T/QRS value. Cases with the same initial baseline T/QRS value were grouped. The intensity of the black colour of the data points relates to the total number of patients that were represented in the group (right column in the graph). The red line represents a linear fit through the data points. The fit suggests a reduction in the pH of 0.0009 and an increase in the base deficit of 0.03 for a rise in 0.1 of the initial baseline T/QRS. The respective correlation coefficients between data points and fit are -0.04 (p = 0.14) and 0.03 (p = 0.34), as calculated with the linear regression model. Abbreviations: BDecf = base deficit in the extracellular fluid, pHart = pH of the arterial cord blood.

In Fig 4, we present the pH of the arterial cord blood and base deficit of the extracellular fluid as a function of the initial baseline T/QRS value. The results show no dependency between pH and base deficit on the one hand, and height of the initial baseline on the other hand. The non-significant correlation coefficient between pH and initial baseline height and between base deficit and baseline height was -0.04 (p = 0.14) and 0.03 (p = 0.34), respectively. These results are in line with the results of Becker et al. [28].

This study suggests that variations in the orientation of the fetal electrical heart axis affect the height of the initial T/QRS baseline and that the height of this baseline determines the occurrence of ST events. This finding could explain for the false ST events that are experienced in everyday clinical practice.

Our aim was to demonstrate that ST events can occur due to normal variations in human physiology (due to variation in electrical fetal heart axis). Therefore, we chose to exclude all cases of metabolic acidosis in this post-hoc analysis. The ST events included in our study, were therefore not related to fetal distress.

The distribution of initial T/QRS baseline values in Fig 2 shows that relatively high baselines are encountered more often than low baselines. Since high baselines are hypothesised to lead to false positive ST events (i.e. alarms while good fetal condition) and low baselines are hypothesised to lead to false negative ST events (i.e. no alarms while compromised fetal condition), this distribution of baseline values can explain why more false positive than false negative ST events are encountered in clinical practice. Since higher baselines do not relate to higher incidences of fetal distress (see Fig 4 and Becker et al. [28]) and considering the large patient population we analysed, we conclude that the presented results support our hypothesis. In other words, some fetuses have a relatively high probability of getting ST events and some fetuses have a relatively low probability, irrespective of their condition. Whether the relatively low probability of getting ST events in case of low initial T/QRS baseline indeed leads to more false negative ST events needs to be confirmed on a dataset including more cases of metabolic acidosis.

In addition, we propose that ST events are unreliable in case of high or low baseline T/QRS values. In case of an average T/QRS baseline value, the incidence of false ST events will be lower. When using the STAN monitor in clinical practice, clinicians should be aware of this limitation. In case of inconclusive cardiotocogram assessment in combination with a high or low baseline T/QRS, fetal blood sampling can be used for complementary diagnostic information. However, the additional value of fetal blood sampling is uncertain and repeated fetal blood sampling is an independent risk factor for caesarean delivery [29]. In case of average baseline T/QRS, ST events can be considered more reliable and could be considered as complementary diagnostic information. ST analysis based on relative elevations of the T/QRS ratio with respect to the baseline or standardised non-invasive fetal ECG recordings [30] might be feasible solutions, that warrant further research. In addition, the relation between signal quality and T/QRS reliability needs to be explored in future research, including analysis of the effects of signal quality of small variations in the ECG that are caused by e.g. rotation of the fetal head at the end of labour.

## Conclusions

This study showed a significant increment of ST events with increasing height of the initial T/QRS baseline; correlation coefficient 0.63, p<0.001. The orientation of the fetal electrical heart axis affects the height of the T/QRS baseline, and therefore the incidence of ST events. This should be taken into account in fetal monitoring with ST analysis.
